# Development and Application of an LC-MS/MS Untargeted Exposomics Method with a Separated Pooled Quality Control Strategy

**DOI:** 10.3390/molecules27082580

**Published:** 2022-04-16

**Authors:** Gianfranco Frigerio, Camilla Moruzzi, Rosa Mercadante, Emma L. Schymanski, Silvia Fustinoni

**Affiliations:** 1 Luxembourg Centre for Systems Biomedicine (LCSB), University of Luxembourg, 6 Avenue du Swing, L-4367 Belvaux, Luxembourg; gianfranco.frigerio@uni.lu (G.F.); emma.schymanski@uni.lu (E.L.S.); 2 Department of Clinical Sciences and Community Health, University of Milan, 20122 Milan, Italy; camilla.moruzzi@studenti.unimi.it (C.M.); rosa.mercadante@unimi.it (R.M.); 3 Occupational Health Unit, Fondazione IRCCS Ca’ Granda Ospedale Maggiore Policlinico, 20122 Milan, Italy

**Keywords:** liquid chromatography tandem mass spectrometry, untargeted metabolomics, pooled quality controls, exposomics

## Abstract

Pooled quality controls (QCs) are usually implemented within untargeted methods to improve the quality of datasets by removing features either not detected or not reproducible. However, this approach can be limiting in exposomics studies conducted on groups of exposed and nonexposed subjects, as compounds present at low levels only in exposed subjects can be diluted and thus not detected in the pooled QC. The aim of this work is to develop and apply an untargeted workflow for human biomonitoring in urine samples, implementing a novel separated approach for preparing pooled quality controls. An LC-MS/MS workflow was developed and applied to a case study of smoking and non-smoking subjects. Three different pooled quality controls were prepared: mixing an aliquot from every sample (QC-T), only from non-smokers (QC-NS), and only from smokers (QC-S). The feature tables were filtered using QC-T (T-feature list), QC-S, and QC-NS, separately. The last two feature lists were merged (SNS-feature list). A higher number of features was obtained with the SNS-feature list than the T-feature list, resulting in identification of a higher number of biologically significant compounds. The separated pooled QC strategy implemented can improve the nontargeted human biomonitoring for groups of exposed and nonexposed subjects.

## 1. Introduction

Following genomics, transcriptomics, and proteomics, metabolomics is the youngest of the “omics” disciplines [1,2]. Defined as the measurement of the metabolic response of living systems to pathophysiological stimuli or genetic modification [3], metabolomics encompasses the comprehensive study of the metabolome, i.e., the ensemble of the small chemical compounds produced from chemical transformations within cells of living organisms [4,5]. The challenge of metabolomics, compared with the other “-omics” sciences, is the chemical complexity and heterogeneity of metabolites [6]. The metabolome is composed of both endogenous metabolites, i.e., metabolites mainly originating from the physiological metabolism, and metabolites originating from exogenous chemicals absorbed by the organism from the environment, including either the original compounds and biotransformation products [7]. Exogenous metabolites are of particular interest as environmental exposures are recognised to play an important role in chronic diseases; thus, the term “exposome” was defined to indicate the totality of exposures an individual is subjected to throughout the lifespan [8]. “Exposomics” is regarded as the comprehensive study of the exposome [9]. Biological monitoring (or biomonitoring) is a useful approach to study the exposome, as it consists of the determination of exogenous compounds, or their metabolites, in biological samples such as urine. The development of analytical methods to comprehensively biomonitor exposure to chemicals remains a current challenge [10]. In particular, such methods can be grouped into targeted and untargeted approaches: while a targeted method aims to accurately quantify a certain number of known compounds, the goal of an untargeted experiment would be to maximise the coverage of detected metabolites as much as possible [11,12]. Untargeted approaches can potentially unravel differences in the metabolic profiles without a priori knowledge, compared with targeted methods. However, a main disadvantage is the reduced ability to detect compounds present at low concentrations, which is especially important for assessing exposure to hazardous chemicals in certain occupational and environmental conditions in the frame of a biomonitoring study [10,13].

Liquid chromatography (LC) coupled with high-resolution mass spectrometry (MS) is a widely used technique to carry out untargeted assays [5,14,15]. To increase the coverage of detected compounds, a possible strategy implemented in untargeted workflows is the combined use of different chromatographic systems, such as reversed-phase liquid chromatography (RPLC) and hydrophilic-interaction liquid chromatography (HILIC), together with the combination of different polarity-ionisation modes in the mass spectrometer (positive and negative) [16,17,18].

The sample preparation for an untargeted approach should be suitable to allow the detection of as many metabolites as possible and to guarantee robustness and reproducibility [19]. Urine is a biofluid that is easy to collect in human biomonitoring studies; it is composed mainly of water along with organic compounds, electrolytes and metabolites [17]. Compared with other biological fluids, it has a lower protein content; therefore, it is not strictly necessary to use organic solvents to denature proteins [20]. To avoid the perturbation and modification of the several metabolites with different physical-chemical properties, usually the sample is kept as unaltered as possible during its preparation: the simple dilution of urine with water and centrifugation before injection is a commonly used strategy [21]. The alternative is to use organic solvents such as acetonitrile or methanol instead of water, which allows the rapid removal of microorganisms and residual proteins from the sample, particularly important in the case of patients subject to proteinuria induced by renal damage [22]. Regardless of the solvent used, the dilution factor applied to the sample is an important consideration, as metabolites co-eluting with more abundant matrix components can suffer from ion suppression. While a higher dilution of the sample is associated with a reduction of in-source ion suppression, less-concentrated metabolites can be diluted below the detection limit [23]; thus, a good compromise must be found.

Following LC-MS analysis, data from the raw mass spectra are usually deconvoluted into a data matrix consisting of samples × metabolite features. For LC-MS, each feature can be defined as a peak with a certain mass-to-charge ratio (*m/z*) and retention time (RT). Such a data matrix can be obtained using computational tools such as XCMS [24,25]. However, with this approach, signals deriving from compounds present in the mobile phases or other contaminants from sample collection, handling, or processing will be present in the data matrix, as well as artefacts generated from the instrument and noise that may be detected by the computational algorithm as actual peaks. To ensure that the data matrix reflects only real biological compounds of interest, an approach is to use a pooled quality control (QC) sample. This is a solution obtained by mixing an equal volume of each test sample, which is then analysed repeatedly throughout the analytical sequence [26,27,28]. Afterwards, the data matrix can be filtered by removing the features not detected or with a low detection rate and with a high relative standard variation in QC analyses, or with a high contribution from blank samples [26]. This approach is widely used within the metabolomics community [29], but presents a limitation for exposomics studies investigating the differences of environmental exposures in cases vs. controls studies, where a group of exposed individuals is compared with a group of unexposed individuals. This is because xenobiotics relevant to exposomics are often at very low concentrations compared with metabolites. Thus, such low-concentrated compounds, uniquely present in samples obtained from exposed subjects, can be diluted in a pooled QC prepared by mixing all samples, eventually not detected in QC samples, and finally filtered from the dataset.

Considering all this, the aim of this study was to develop an analytical method suitable for the untargeted analysis of urine samples, with a particular focus on exogenous metabolites deriving from environmental exposures. In particular, a differentiated strategy for pooled QCs is proposed by preparing separated QCs for each group in the study and compared with the single-pool-sample approach in the context of smoking/non-smoking subjects.

## 2. Results

### 2.1. Optimisation of the Sample Preparation

**Figure 1:**
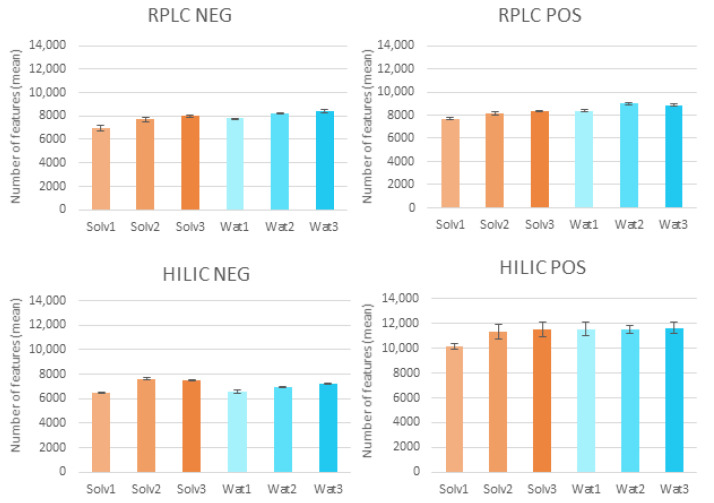
Bar plots representing the number of features (y axis) for each analytical condition (reverse-phase liquid chromatography, RPLC, and hydrophilic-interaction liquid chromatography, HILIC) with negative (NEG) and positive (POS) ionisation mode (x axis), and within a plot, different sample preparations (solvent or water-sample dilution). Each bar represents the mean number of features of three different replicates, and the error bars represent the standard deviations. Solv1: 250 µL of sample and 750 µL of methanol:acetonitrile; Solv2: 500 µL of sample and 500 µL of methanol:acetonitrile; Solv3: 750 µL of sample and 250 µL of methanol:acetonitrile; Wat1: 250 µL of sample and 750 µL of water; Wat2: 500 µL of sample and 500 µL of water; Wat3: 750 µL of sample and 250 µL of water.

Figure 1 shows the differences in numbers of features obtained with the different sample preparations tested, as a mean of the three replicates performed for each solvent or dilution tested. The features ranged from 6479 to 11,646 and generally did not vary greatly among the different experiments, although a greater number of features can be observed in less-diluted samples. A greater number of features was observed in Solv2 than Solv1, while this difference was less evident between Solv3 and Solv2. For this reason, the intermediate dilution factor (Solv2) was chosen for further experiments. While the use of pure water led to a higher number of features in RPLC POS, the organic solvent mixture was chosen instead to be sure of removing residual proteins for patients with kidney damage and to prevent microbial growth in the sample. Figure S1 shows the distribution of the coefficient of variation, calculated for features only present in all samples considered over the three experiments for each combination of solvent or dilution tested. Most features were reproducible, with coefficient of variations (CVs) <20%.

### 2.2. Analysis of the Analytical Standards

The analytical standards analysed were manually screened with Sciex OS, with the results reported in Supplementary Table S1.

### 2.3. Application to Unknown Samples: Different Quality Control Approaches

**Figure 2:**
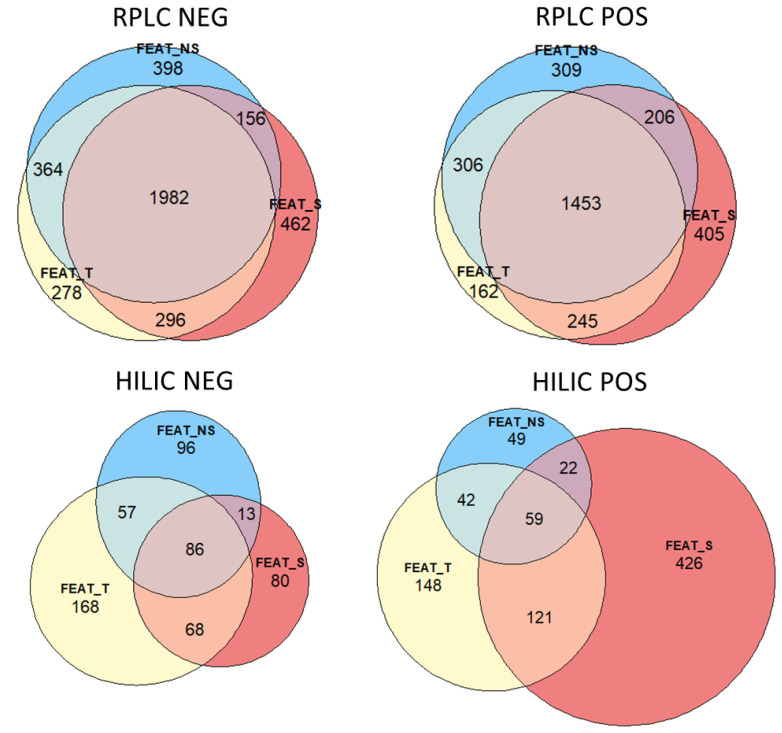
Eulero–Venn graphs, for each chromatographic run, showing the differences in the number of features obtained by separately filtering the dataset with the QC-T (quality controls from all samples) (FEAT_T), the QC-S (quality control from smokers) (FEAT_S), or the QC-NS (quality control from non-smokers) (FEAT_NS).

The Eulero–Venn graphs in Figure 2 show the differences in the number of features obtained using the QC-T (quality controls from all samples), QC-S (quality control from smokers), or QC-NS (quality control from non-smokers) filtration approaches. For RPLC NEG, the total number of features obtained after processing with XCMS was 27,538. Filtering these features considering QC-T, i.e., removing features either not detected or not reproducible in the QCs or with a high signal in the blank (see Section 4.5), reduced this to 2920 features, whereas 2900 features were kept considering QC-NS and 2896 with QC-S. Merging the last two feature lists resulted in a SNS-feature list containing 3658 unique features. For RPLC POS, 18,855 total features were reduced to 2166 QC-T features, 2274 QC-NS features, 2309 QC-S, and a merged SNS-feature list of 2924 unique features. For HILIC NEG, 18,904 features were obtained with XCMS. This was reduced to 379 QC-T, 252 QC-NS, 247 QC-S features, and a merged SNS-feature list of 400 unique features. For HILIC POS, 18,103 total features were filtered to 370 QC-T features, 172 QC-NS and 628 QC-S features, and a merged SNS-feature list of 719 unique features.

**Figure 3:**
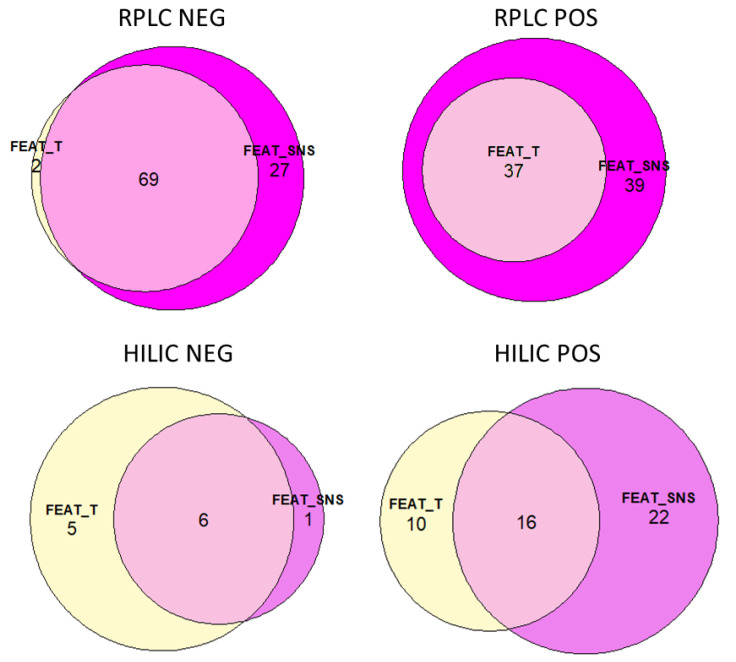
Eulero–Venn graphs for each chromatographic run, showing the differences in the number of statistically different features between the group of smoking subjects and the group of non-smoking subjects, when considering the T-feature list (FEAT-T) or the SNS-feature list (FEAT-SNS).

For each of the four chromatographic runs, a *t*-test was applied to find statistically significant features between non-smokers and smokers. This was applied separately to both the T-feature list and to the SNS-feature list. The Eulero–Venn graphs reported in Figure 3 show the differences in numbers of significant features obtained from the different feature lists for each chromatographic run. For RPLC NEG, 71 features were significantly different between groups in the T-feature list, while 96 were significantly different with the SNS-feature list. Comparing the results, 69 were in common between the two lists, while 27 were unique in the SNS-feature list and 2 were from the T-feature list. For RPLC POS, 37 features were significantly different between groups in the T-feature list, while 76 were significantly different with the SNS-feature list. All 37 significant features in the T-feature list were also in the SNS-feature list, while the remaining 39 were unique to the SNS-feature list. For HILIC NEG, 11 features were significantly different between groups in the T-feature list, while 7 were significantly different with the SNS-feature list. Of these, 6 were in common, 1 was unique to the SNS-feature list and 5 to the T-feature list. Finally, for HILIC POS, 26 features were significantly different between groups in the T-feature list, while 38 were significantly different with the SNS-feature list. Of these, 16 were in common, 22 were unique to the SNS-feature list, and 10 to the T-feature list.

**Figure 4:**
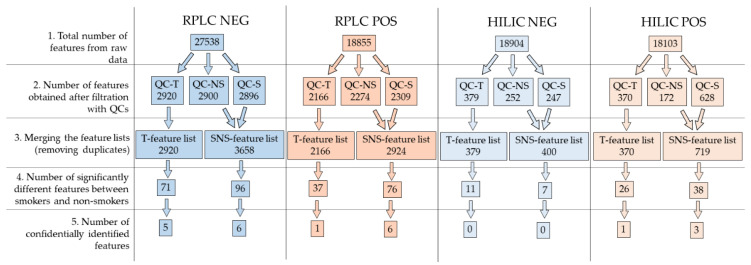
Summary of the data elaboration, for each of the four analytical conditions. 1: Total number of features extracted from raw data using the XCMS algorithm. 2: The total feature list was filtered by discarding features present in less than 50% of the QCs, those with a CV ≥ 30% among QCs, and with a blank contribution ≥ 10%. This procedure was performed separately using QC-T, QC-NS, and QC-S. 3: The feature lists obtained using QC-NS and QC-S were merged by eliminating duplicates, to yield the SNS-feature list. 4: A *T*-test was performed to find significantly different features between urine samples from smokers and non-smokers; this was performed separately with the T-feature list and with the SNS-feature list. 5: Number of features confidentially identified (level 1) with external analytical standards under the same analytical conditions.

The identity of the unknown features was investigated by comparison with the analytical standards. In total, nine unique compounds could be identified confidently (i.e., via comparison with an external analytical standard analysed in the same condition as the unknown samples) across all samples and analytical conditions. These are reported in Table 1. For RPLC NEG, of the 98 significantly different features, 6 were confidently identified. 2-Hydroxypropylmercapturic acid (2-HPMA) was retrieved only within the SNS-feature list. For RPLC POS, of the 76 significantly different features, 6 could be confidently identified. Of these, five—nicotine and an isotope of it, N-Acetyl-S-(3,4-dihydroxybutyl)-L-cysteine (DHBMA), N-Acetyl-S-(3-hydroxy-1-methylpropyl)-L-cysteine (HMPMA), and phenylglyoxylic acid—were retrieved only within the SNS-feature list. None of the 12 HILIC NEG features could be identified via analytical standards. For HILIC POS, 3 of the 48 significantly different features were confidently identified. The compounds 3-benzoylpropionic acid and HMPMA were retrieved only within the SNS-feature list. A further description of how the features were filtered in all steps and for all four analytical conditions is given in Figure 4.

## 3. Discussion

This work describes a comprehensive untargeted method, useful for the human biomonitoring of potentially unknown compounds. The sample preparation, the chromatographic and mass spectrometry conditions, and in particular, the pooled quality control strategy were developed taking into consideration the challenges of studying compounds and metabolites originating from environmental exposures. The aim of the developed workflow was to provide a suitable approach to characterise the exposome in groups with different exposures.

Several efforts were made for the development of the chromatographic methods. In order to maximise the coverage of metabolites, the intent was to perform both RPLC and HILIC. A literature search revealed the results reported by Contrerpois et al., where they investigated the performance of five different HILIC columns, operating at three different pH (acidic, neutral, basic) and five C18 RPLC columns by testing both a set of known metabolites (and checking the retention time, peak shape, and MS signal), and unknown ones (with the aim of maximising the number of detected metabolic features). Overall, according to the results reported by Contrerpois et al., zwitterionic column ZIC-HILIC at neutral pH and the RPLC column Hypersil GOLD were the columns with the best results [16] and were thus chosen for this study. Regarding the optimisation of the chromatographic conditions, efforts included optimising all the parameters by comparing the response for known compounds (in terms of signal-to-noise, area-to-height ratio, peak width at 50% of the height) and the number and reproducibility of unknown features, for the different parameters tested. In particular, different mobile phases were tested for RPLC: water was always used as the aqueous phase, while both acetonitrile and methanol were tested as the organic phase. Although acetonitrile gave better results in terms of peak efficiency, methanol was chosen due to the greater number of features obtained. The use of acidic modifiers was also tested comparing acetic acid, formic acid, and a solution with 10 mM ammonium acetate and acetic acid 0.1% (pH 6). As a significant decrease in the signal with the buffer and with formic acid was observed for some of the known compounds, we decided to implement acetic acid. Furthermore, for RPLC, a comparison of two different flow rates was carried out: both 200 and 300 μL/min were tested, and the higher flow rate was finally implemented as the overall retention times were lower while still keeping a good separation between the peaks. For HILIC, the decision for the mobile phases were based on the previous experiments conducted by Contrepois et al. [16], while different gradients were tested. Moreover, different injection volumes were tested (2, 3, 4, and 5 µL) for HILIC: a higher number of features was obtained with the higher injection volume tested (5 µL), without compromising peak efficiency.

In general, one of the biggest bottlenecks in metabolomics or exposomics investigations is the identification of unknown compounds [15]. Researchers are encouraged to assign a “level of confidence” to each identified/annotated compound and the highest level of confidence is achieved when a compound has the same retention time and mass fragmentation pattern of an analytical standards analysed with the same instrument and under the same conditions [30,31]. For this reason, we collected from our laboratory as many analytical standards as possible in order to have as many potential level 1 s as possible, thus increasing the likelihood of findings with the highest level of confidence while analysing unknown samples. The use of these standard mixtures was also useful for method development, and further confirmed the necessity of analysing samples with four different runs (RPLC NEG, RPLC POS, HILIC NEG, and HILIC POS) as some of these compounds responded properly only under certain condition.

The experiments for the optimisation of the sample preparation were set up considering mainly the work published by Southam and coworkers, which provided solid results for the reproducible and high-throughput metabolic phenotyping [22]. However, there were concerns relating to the possible overdilution of the sample, as maximum possible sensitivity of the method was desired. Therefore, since Southam et al. tested several solvents but all with a dilution 3:1, this work extended these efforts by testing several dilution factors, and as reported in Section 4.2, found that the dilution 1:1 could be a good compromise mediating the dilution factor and the possible in-source interference related to the matrix effects. Finally, based on both the results described above and the ones reported by Southam et al., the same sample-preparation method was chosen for both the RPLC and HILIC chromatography. This can be useful both for consistency of results obtained, and also to ensure the high-throughput nature of the entire workflow, as the contents of the same vials can be injected in the four different chromatographic conditions.

The main novelty of this workflow was how the pooled QC samples were prepared. The results presented here demonstrate the advantage of preparing separated pooled QC samples for exposomics investigations—one for each group of exposure—and subsequently filtering the features using information from both pooled QCs and then merging the feature lists. This allows for better cleaning of the feature lists without the risk of losing metabolites of interest, in particular those present only in the exposed group of subjects, which can possibly be diluted and not detected in a pooled QC prepared from all samples. Indeed, this approach revealed a higher number of statistically significant features. The Eulero–Venn diagrams reported in Figure 2 and Figure 3 are useful to understand the differences among the different chromatographic runs. Indeed, Figure 2 shows that a high number of features was in common among all the three feature lists for RPLC NEG and POS, while for HILIC POS and NEG, there was a greater difference. Apart from HILIC NEG, the number of features uniquely present in FEAT-S was higher than the number of unique features in FEAT-NS and FEAT-T; this can be caused by the presence of a greater number of compounds derived from smoke. Figure 3 highlights that the advantage of considering the SNS-feature list was especially successful for RPLC NEG and POS, as most of the significant features were from the SNS-feature list (see the summary reported in Figure 4). 

The results demonstrate that implementing separated pooled QCs and merging the filtered feature lists obtained with the pooled QCs from exposed and from nonexposed subjects (the SNS-feature list) increases the number of biologically relevant compounds detected. An additional option for future applications is also to combine the T-feature list with the SNS-feature list, to obtain an even greater feature list; for the application reported in this paper, such an “SNST-feature list” would be composed of 3936 features for RPLC NEG (278 unique from the T-feature list), 3086 features for RPLC POS (162 unique from the T-feature list), 568 for HILIC NEG (168 unique from the T-feature list), and 867 for HILIC POS (148 unique from the T-feature list). However, none of the confidentially identified compounds of our experiments derived from the unique T-feature list, and the investigation of the remaining unknown features is beyond the scope of this article.

Some previously published approaches reported the use of differently prepared pooled QC samples, although with different purposes or applying different data-processing techniques. Guy et al. used two different QC samples for urine: one prepared from in-house urine, not belonging to the population of interest, and spiked with standards of interest, while the other was prepared taking an aliquot from each study sample; in this approach the QCs were used to align retention time, mass precision, and ion intensities [32]. Zhang and coworkers proposed a pooled strategy consisting of generating two or more pooled samples for each sample group, than background-subtracting the data from the treated pooled samples from the control pooled samples, or vice versa [33]. Hsu et al. conducted different experiments preparing separated pooled quality controls for blood samples taken from smokers and non-smokers, and used them to test variability and reproducibility of the assays [34]. Go and coworkers also (as in this study) performed biomonitoring of environmental chemicals present in a small fraction of people by proposing to use pooled reference samples with known relevant concentrations of chemicals of interests, i.e., a “reference standardisation” protocol, where the concentrations of compounds in unknown samples are estimated by comparison to a pooled reference sample with known chemical concentrations [35]. Shen and coworkers suggested, for exposomics analyses, to prepare and analyse the data from the pooled samples (by preparing at least five pools with 30 samples per pool from the case and control populations). This approach has the advantage of being very convenient for reducing costs, workload, and the need for large sample volumes, but has the disadvantage of losing individual information [36], while this is something still possible with the approach developed in this work. In general, if compared to the other strategies reported in the literature, the advantage of our work is the direct comparison between the total QC strategy and the separated QC strategy, in terms of number of features obtained and confidentially identified compounds.

The effectiveness of the approach proposed here was further proven with the compounds confidently identified, thanks to the pool of the analytical standards analysed. Indeed, several features belonging to mercapturic acids were identified only using the proposed differentiated QC pooled-sample approach proposed. Mercapturic acids are sensitive metabolites of volatile organic compounds and were already measured in this population with a targeted approach, demonstrating that they are significantly higher in smoking subjects [37]. Nicotine and its metabolite, cotinine, are important compounds which differentiate between smoking and non-smoking subjects [38]. Interestingly, while cotinine was detected also with the traditional QC approach, nicotine was only detected using the separated QC approach here proposed. Phenylglyoxylic acid is another metabolite identified thanks to the proposed approach, which is a metabolite of styrene [39]. Overall, 15 significantly different features were confidently identified, among which 8 were kept after QC filtration only when using the separated pooled QC strategy proposed in this work. It is not surprising that only these few compounds were identified as significantly different between groups out of the 139 chemical standards considered, as the standards collected in our laboratory included also endogenous metabolites and molecules related to occupational exposures or to exposure to solvents or pesticides, not expected as different among the healthy subjects considered in this case study. Furthermore, as previously reported [13], compounds present at very low concentrations (such as the specific metabolites of benzene and 1,3-butadiene) were still not detected with this untargeted measurement.

Despite the advantage of reducing the loss of compounds of interest, the proposed pooled QC strategy does reduce the performance of the method slightly compared with previous approaches, since preparing more pooled QC samples and analysing them increases the time required for sample preparation and the duration of the sequence. However, this can be considered a minor limitation, as the main bottleneck in untargeted metabolomics is the identification of metabolites [15], and the additional time required for this preparation can improve it, as demonstrated in this work. Another limitation is that there was no noticeable improvement for HILIC NEG using this separated approach. However, as shown with the results here reported comparing smoking with non-smoking subjects, the separated pooled QC strategies can be useful for exposomics investigations aimed at the detection of unknown compounds in a specific exposed population compared to a reference nonexposed population. This enabled the development of a valid workflow, allowing to both filter those signals not of biological interest, and at the same time, to avoid filtering potential compounds of interest.

In conclusion, the developed method is a valid nontargeted approach for exposomic assays. Even though untargeted assays may lack sensitivity, the efforts made for this work demonstrated improvements in the number of confidently identified compounds in nontargeted measurements when using the separated quality control strategy. The separated quality control strategy implemented and applied to the data interpretation is the main novelty of the present work and can further help in improving sensitivity of untargeted investigations while also guaranteeing a suitable quality of the dataset.

## 4. Materials and Methods

### 4.1. Study Subjects, Chemicals, and Standard Solutions

A total of 60 urine samples were collected from 38 non-smoking subjects (NS) and 22 smoking subjects (S). The samples were collected in the frame of an occupational monitoring campaign, which was described previously [37].

A total of 144 standards, including both analytical standards and standard mixtures taken from commercial kits available in the laboratory, were collected. The complete details of the purchased standards are given in the Supplementary Materials (Supplementary Text). Due to the occurrence of duplicates among pure standards and commercial kits, the number of unique standards was 139.

Eleven urine samples were collected from healthy volunteers, including five females and six males. These aliquots were mixed, and the resulting solution was used for the dilution of standard solutions and for the optimisation of the sample preparation and chromatographic conditions. Details of the preparation of the standard solutions are given in the Supplementary Materials.

### 4.2. Sample Preparation

#### 4.2.1. Comparison of Different Sample Preparations

Southam et al. tested different solvent mixtures and incubation conditions for the preparation of urine samples for untargeted clinical metabolic phenotyping, reporting that monophasic 50:50 methanol:acetonitrile with no incubation is good option in terms of reproducibility, putative metabolite count, and total peak area [22]. In their experiment, Southam et al. did not test different dilution factors, as they dilute 50 µL of urine in 150 µL of the tested solvent or solvent mixture (1:3), and did not test the use of pure water. Thus, additional experiments were performed here to evaluate the ideal dilution factor and to test the use of water. In particular, the urine sample was diluted with the methanol:acetonitrile mixture in three different ways: 250 µL of the urine sample with 750 µL of methanol:acetonitrile (1:3) (Solv1), 500 µL of the urine sample with 500 µL of methanol:acetonitrile (1:1) (Solv2), and 750 µL of the urine sample with 250 µL of methanol:acetonitrile (3:1) (Solv3). Furthermore, the urine sample was diluted with water with the same dilution factors used for methanol:acetonitrile: 250 µL of the urine sample with 750 µL of water (1:3) (Wat1), 500 µL of the urine sample with 500 µL of water (1:1) (Wat2), 750 µL of the urine sample with 250 µL of water (3:1) (Wat3). For the purpose of this experiment, each dilution was prepared in triplicate, and a blank sample of 1000 µL methanol:acetonitrile was also prepared. The samples were then centrifuged at 10,900 r.p.m. (10,500× *g*) for 20 min, then the supernatant was transferred to an autosampler vial.

#### 4.2.2. Comparison of Two Strategies for Preparing QC Samples

Since, in the frame of an exposomics study, the molecules present at very low concentrations only in the exposed group can be diluted and not detected in a total pooled QC, this work also investigated the use of pooled QCs prepared separately from each exposure group. In particular: an aliquot of 150 µL from all samples were mixed to obtain the total pooled QC (QC-T), whereas aliquots of 250 µL from samples collected from non-smoking subjects were mixed to obtain a non-smoker pooled QC (QC-NS) and aliquots of 450 µL from samples collected from smoking subjects were mixed to obtain a smoker pooled QC (QC-S).

Afterwards, the analytical standard solutions and the 60 samples from study subjects were prepared: 500 µL of the sample (unknown samples, or pooled QC, or analytical standard mixture, or pure water as blank sample) was added to 500 µL acetonitrile:methanol 1:1, vortexed, and centrifuged for 15 min at 10,900 r.p.m. (10,500× *g*). The supernatant was then transferred to an autosampler vial and analysed.

### 4.3. LC-MS/MS Analyses

The analyses were performed with ultra-high-pressure liquid chromatography (UHPLC) coupled to time-of-flight (TOF) tandem mass spectrometry (MS/MS). To increase the metabolite coverage, every sequence was analysed in four different runs, corresponding to the combination of two different chromatographic separations, and two polarity (ion) modes, negative or positive. The UHPLC system was an Exion LC (AB Sciex, Monza, Italy); the autosampler temperature was set at 10 °C, and the injection volume was set to 2 µL for RPLC and 5 µL for HILIC. The column used for RPLC was a Hypersil GOLD, C_18_ (100 × 2.1 mm; 1.9 μm; 175 Å; Thermo Fisher Scientific, Rodano, Italy) with a guard-column SecurityGuard^TM^ C_18_ (4 × 3 mm; Phenomenex, Castel Maggiore, Italy). The column used for HILIC was a ZIC HILIC, Sulfobetaine (100 × 2.1 mm; 3.5 μm; 200 Å; Merck, Darmstadt, Germania), with a guard-column ZIC HILIC, Guard Kit (20 × 2.1 mm; Merck, Darmstadt, Germania). The column was kept at 50 °C for both HILIC and RPLC. Linear gradients with two mobile phases were developed and optimised. For RPLC, the A phase was acetic acid 0.1% in water, B phase was 0.1% acetic acid in methanol, and the gradient program was set up as follows: initial step 1% B; 0–1 min, 1% B isocratic; 1–8 min, from 1% to 100% B; 8–12 min, 100% B isocratic; 12–12.1 min, from 100% to 1% B; 12.1–18 min, 1 % B isocratic. The flow was kept constant at 300 µL/min. For HILIC, the A phase was ammonium acetate 10 mM in acetonitrile:water 95:5 (*v*/*v*) (pH 5.5), the B phase was ammonium acetate 10 mM in acetonitrile:water 5:95 (*v*/*v*) (pH 6), and the gradient program was set up as follows: initial step 1 % B; 0–0.5 min, 1 % B isocratic; 0.5–5 min, from 1% to 30% B; 5–6 min, from 30% to 100% B; 6–8 min, 100% B isocratic; 8–8.1 min, from 100% to 1% B; 8.1–16 min, 1% B isocratic. The flow was kept constant at 400 µL/min. The mass spectrometer was a Triple-TOF 6600 (AB Sciex, Monza, Italy) in data-dependent mode: for each scan cycle, a full mass experiment (50–450 *m/z*) was conducted, followed by the MS/MS fragmentation of the top 20 most intense signals, considering only signals with an intensity of at least 1000 count per seconds (cps) and excluding the same parent ion for 10 s after two consecutive triggers. Electrospray ionisation (ESI) source was used with the following parameters: curtain gas (N_2_), 30 psi; collision gas, low; ion spray voltage, ±4500 V; temperature, 350 °C; ion source gas 1 (air), 50 psi; ion source gas 2 (air), 45 psi; declustering potential, ±50 V; collision energy, ±15 V with a collision energy spread of 30 V. During each analytical sequence, an external calibration was performed every three analyses, according to the manufacturer’s instructions, to calibrate the mass accuracy of the spectrometer. Data were acquired in the high-resolution mode (HR). The Analist^®®^ software (version 1.7.1; Ab Sciex S.r.l, Milano, Italy) was used to prepare batches for analysis.

### 4.4. Analytical Sequence

A blank sample (water prepared as the samples) and series of pooled QCs (including QC-T, QC-NS, and QC-S) were analysed at the beginning of the sequence (data later discarded during data processing), followed by a blank sample and an additional series of QCs. Then, the unknown samples were injected in a random order, interspersed with the QCs. At the end of the sequence, a final series of the QCs followed by a blank sample were analysed. The sequence was repeated four times for each of the following modes: RPLC negative-ion mode (RPLC NEG), RPLC positive-ion mode (RPLC POS), HILIC negative-ion mode (HILIC NEG), and HILIC positive-ion mode (HILIC POS).

### 4.5. Data Integration, Analysis, and Metabolite Annotation

The data obtained from the analysis were processed using a combination of vendor software and open-source tools. The vendor software Sciex OS was used to manually search for the analytical standards included in the analyses. Data were also converted from the “wiff” to the “mzML” format using ProteoWizard MSConverter (version 3.0.20029) [40,41] using the vendor peak picking algorithm on all MS levels. The software R (version 4.0.2) [42] was used for most of the following processing, via the RStudio interface (version 1.3.959, RStudio Inc., Boston, MA, USA) and the “tidyverse” package [43]. The files—in particular the pooled QCs—were then processed with the IPO algorithm [44] to optimise input parameters for XCMS, which was used to obtain feature lists [24,25]. The XCMS parameters were manually adjusted, as the modified parameters were able to obtain a higher number of reproducible features. The following parameters were used for all four chromatographic/polarity modes: ppm, 30; mzdiff, −0.012; bw, 10; mzwid, 0.0265; gapInit, 0.5; gapExtend, 2; minfrac, 0. For RPLC data (both positive and negative mode) the min and max peak width were 17.376 and 54.68, respectively; for HILIC negative they were 28 and 65, while for HILIC positive they were 9.92 and 28.5. An “xcmsSet”, a “group”, a “retcor”, and another “group” was performed with XCMS to obtain the final peakTable (per mode).

Each of the four tables of features obtained from XCMS was then filtered as follows: To obtain the “T-feature list”, features present in less than 50% of the QC-T, those with CV ≥ 30% among QC-T, and with a blank contribution ≥ 10% (calculated as the percent ratio between mean intensity of the peak in the blank samples and the mean intensity of the peak in QC-T) were discarded. To obtain the S-feature list and the NS-feature list, the same process was performed independently using QC-S and QC-NS. Finally, the S-feature list and the NS-feature list were merged to form the “SNS-feature list”.

A *t*-test was applied to each of the two different feature lists (T-feature list and SNS-feature list) to find significantly different features between non-smokers and smokers. The *t*-test was applied using MetaboAnalyst [45]. Before performing the test, feature intensities were sum-normalised and log-transformed. A multiple testing was performed [46] and a false discovery rate (FDR) *p*-value less than 0.05 was considered statistically significant.

The BEAMS software developed at the University of Birmingham (Birmingham mEtabolite Annotation for Mass Spectrometry) [47] was used to group signals for possible adducts and isotopes, while annotation was conducted only on features that were statistically different according to the *t*-test. Using Sciex OS, features were manually searched in the IDA explorer and MS/MS fragments were extracted and compared to the results obtained from the analyses of analytical standards, to confidentially identify compounds.

The processing of the data for optimising the sample preparation was performed slightly differently: the XCMS parameters were directly set as suggested by IPO. Next, the feature tables were filtered by eliminating the features not present in all samples, those containing missing data, and those with a high blank contribution (percentage ratio between the average intensity in the pure solvents and that of the samples <10%).

## Figures and Tables

**Table 1:** List of confidentially identified features, for each chromatographic run: RPLC NEG (out of 98 significant features), RPLC POS (out of 76 significant features), and HILIC POS (out of 48 significant features). No compounds were confidently identified in HILIC NEG (out of 12 significant features). For each feature, the mass-to-charge (*m/z*) ratio, retention time (RT), and CAS number are reported, along with the FDR *p*-value of the *t*-test, in which group of subjects the compound was higher (smokers, S, or non-smokers, NS), and in which feature lists that feature was present.

**RPLC NEG**						
***m/z***	**RT (min)**	**Molecule**	**CAS Number**	**FDR *p*-Value**	**Difference**	**Presence in Feature Lists**
220.0642	3.55	3-HPMA	23127-40-4	2.52 × 10^−8^	S > NS	Both in SNS and in T
222.0626	3.57	2-HPMA	923-43-3	5.02 × 10^−7^	S > NS	Only in SNS
234.0800	4.47	HMPMA	33164-64-6	7.4 × 10^−7^	S > NS	Both in SNS and in T
235.0813	4.47	HMPMA [^13^C isotope]		1.13 × 10^−3^	S > NS	Both in SNS and in T
215.0016	6.67	CEMA	74514-75-3	1.36 × 10^−3^	S > NS	Both in SNS and in T
216.0044	6.67	CEMA [^13^C isotope]		9.8 × 10^−4^	S > NS	Both in SNS and in T
**RPLC POS**						
***m/z***	**RT**	**Molecule**	**CAS Number**	**FDR *p*-Value**	**Difference**	**Presence in Feature Lists**
177.1011	3.60	Cotinine	486-56-6	8.32 × 10^−24^	S > NS	Both in SNS and T
163.1214	2.26	Nicotine	54-11-5	6.23 × 10^−12^	S > NS	Only in SNS
164.1243	2.26	Nicotine [^13^C isotope]		2.07 × 10^−6^	S > NS	Only in SNS
252.0886	2.04	DHBMA	144889-50-9	7.71 × 10^−5^	S > NS	Only in SNS
236.0941	4.55	HMPMA	33164-64-6	0.039	S > NS	Only in SNS
151.1212	3.88	Phenylglyoxylic acid	611-73-4	0.045	S > NS	Only in SNS
**HILIC POS**						
***m/z***	**RT**	**Molecule**	**CAS Number**	**FDR *p*-Value**	**Difference**	**Presence in Feature Lists**
177.1019	1.05	Cotinine	486-56-6	1.07 × 10^−15^	S > NS	Both in SNS and T
179.0799	1.48	3-Benzoylpropionic acid	2051-95-8	2.64 × 10^−7^	S > NS	Only in SNS
236.0933	4.02	HMPMA	33164-64-6	4.59 × 10^−6^	S > NS	Only in SNS

Detailed information about the compounds is reported in Supplementary Table S1.

## Data Availability

The data presented in this study are available on request from the corresponding author.

## Supplementary Materials

The following supporting information can be downloaded at: https://www.mdpi.com/article/10.3390/molecules27082580/s1, Figure S1: Box plot representing the distributions of the percentage coefficients of variation of feature intensities, for each sample-preparation experiment and for each chromatographic run. The coefficients are calculated only from among features present in every tested solution. Boxplots are built as follows: the median divides the box in two areas and the upper and lower hinge represent the 25th and 75th percentile of the distribution, respectively. Outside the box, the upper and lower whiskers extend from the hinge to 1.5 times interquartile range (IQR). Data beyond the whiskers are plotted individually and represented as dots; Supplementary Text: complete description of the purchase and preparation of the analytical standards; Table S1: Results from the analyses of the analytical standards. Molecules are grouped in the different mixtures prepared (or commercial kit mixtures), and the CAS number is reported, along with the molecular formula and monoisotopic masses (M, [M−H]^−^, [M+H]^+^). For each chromatographic run, if the compound was detected, the ppm error is reported, along with the retention time (RT) and the MS/MS fragmentation patterns (fragment 1, fragment 2, fragment 3). Furthermore, additional information such as PubChem CID, Preferred name, InChIKey, IUPAC name, SMILES, and InChI are also provided in an additional sheet.
