# Supplementary material for: Development and Application of an LC-MS/MS Untargeted Exposomics Method with a Separated Pooled Quality Control Strategy
Source: Molecules. 2022 Apr 16;27(8):2580. doi: 10.3390/molecules27082580 (PMC9031529; doi:10.3390/molecules27082580)
Supplement: Supplementary file 1 [file molecules-27-02580-s001.zip › 2022-03-01_untarg_method_sepQC_Supp_material-FigS1_and_text.pdf]

Supplementary Materials

Development and application of an LC-MS/MS untargeted exposomics method with a separated pooled quality control strategy

Gianfranco Frigerio [1,2,3], Camilla Moruzzi [2], Rosa Mercadante [2], Emma L. Schymanski [1], and Silvia Fustinoni [2,3]*

[1] Luxembourg Centre for Systems Biomedicine (LCSB), University of Luxembourg, 6 avenue du Swing, L-4367 Belvaux, Luxembourg
[2] Department of Clinical Sciences and Community Health, University of Milan, Italy
[3] Occupational Health Unit, Fondazione IRCCS Ca' Granda Ospedale Maggiore Policlinico, Milan, Italy
* Correspondence: silvia.fustinoni@unimi.it

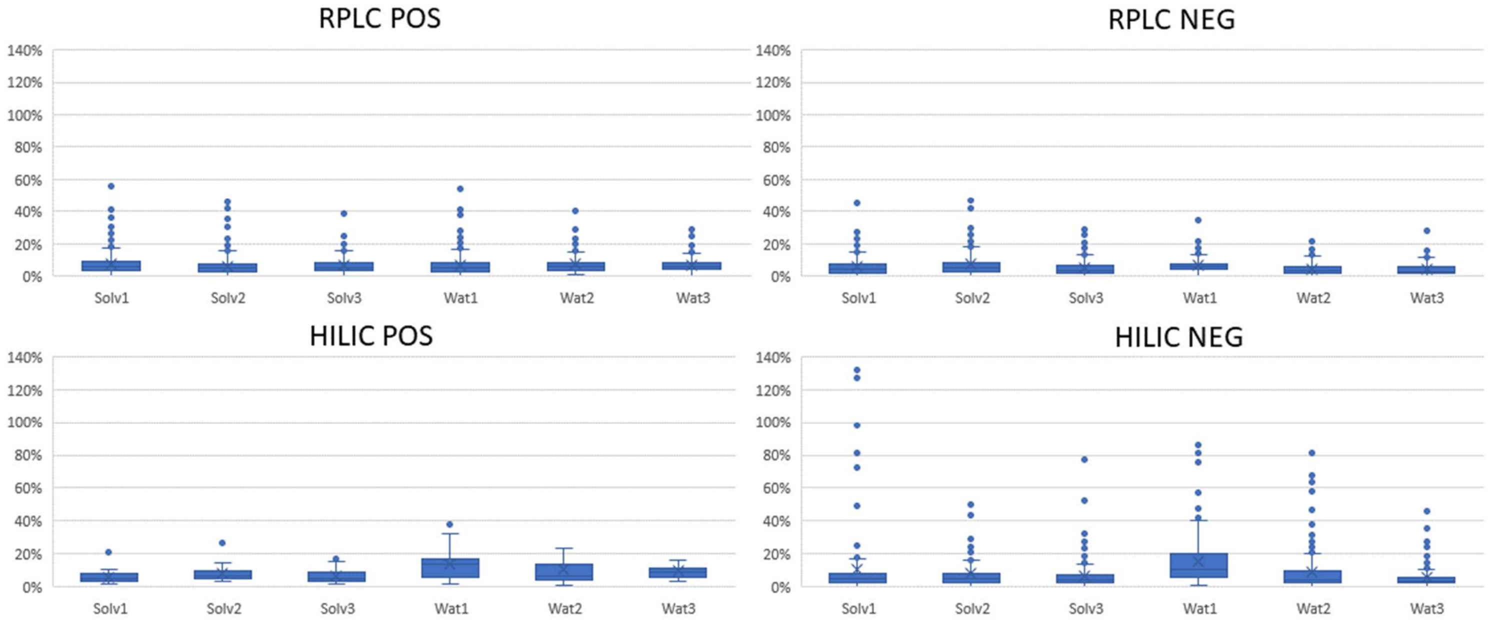

Figure S1: Box plot representing the distributions of the percentage coefficients of variation of feature intensities, for each sample preparation experiment and for each chromatographic run. The coefficients are calculated only from among features present in every tested solution. Boxplots are built as follows: the median divides the box in two areas and the upper and lower hinge represent the 25th and 75th percentile of the distribution, respectively. Outside the box, the upper and lower whiskers extend from the hinge to 1.5 times interquartile range (IQR). Data beyond the whiskers are plotted individually and represented as dots.

Analytical standards

Standards used for "Standard mixture A"

- 8-hydroxyguanosine 100% (Calbiochem, Darmstadt, Germany)
- 8-hydroxy-2'-deoxyguanosine ≥98% (TLC) (Sigma-Aldrich, Steinheim, Germany)
- 8-hydroxyguanine ≥90% (Sigma-Aldrich, Steinheim, Germany)

Standards used for "Standard mixture B"

- 1-hydroxypyrene 98% (Sigma-Aldrich, Steinheim, Germany)
- 1-naphthol, analytical grade for analysis (Merck Darmstadt, Germany)
- 3,5,6-trichloro-2-pyridinol (PESTANAL®) analytical standard (Merck Darmstadt, Germany)
- (-)-cotinine ≥98% (Sigma-Aldrich, Steinheim, Germany)
- (-)-nicotine tartrate salt ≥98% (TLC) (Sigma-Aldrich, Steinheim, Germany)

Standards used for "Standard mixture C"

- 2-naphthol, analysis grade (Merck Darmstadt, Germany)
- Aldosterone ≥95% (HPLC) (Sigma-Aldrich, Steinheim, Germany)
- Hydrocortisone ≥98% (HPLC) (Sigma-Aldrich, Steinheim, Germany)
- Cortisone ≥98% (Sigma-Aldrich, Steinheim, Germany)
- Dexamethasone (Decadron®) (I. B. N. Savio, Pomezia, Italy)
- Prednisone ≥98% (Sigma-Aldrich, Steinheim, Germany)
- Prednisolone ≥99% (Sigma-Aldrich, Steinheim, Germany)

Standards used for "Standard mixture D"

- *Trans,trans*-muconic acid 98% (Aldrich-Chemie, Steinheim, Germany)
- Ethylglucuronide> 99% (Medichem, Steinenbronn, Germany)
- Ethylentiourea 98% (Aldrich-Chemie, Steinheim, Germany)
- Ethyl sulfate> 98% (TCI, Boereveldseweg, Belgium)

Standards used for "Standard mixture E"

- Urea (Fine Chemicals, Milan, Italy)
- 1,3-dimethyl-2-thiourea 99% (Aldrich-Chemie, Steinheim, Germany)
- Propylentiourea 94% (Dr. Ehrenstorfer, Ausburg, Germany)
- Thiourea, ≥99.0%, ACS reagent (Merck, Darmstadt, Germany)
- Benzoic acid 99%, ACS (Aldrich-Chemie, Steinheim, Germany)
- 3-methyluric acid ~ 95% (TLC) (Sigma-Aldrich, Steinheim, Germany)
- 1,7-Dimethyluric acid ~ 95% (HPLC) (Sigma-Aldrich, Steinheim, Germany)

Standards used for "Standard mixture F"

- 3,4-Dimethilippuric acid >98.0% (HPLC) (TCI, Boereveldseweg, Belgium)
- 3-Benzoylpropionic acid >98.0% (GC) (TCI, Boereveldseweg, Belgium)
- 5-(N-benzoylamino)-n-valeric acid >98.0% (GC) (TCI, Boereveldseweg, Belgium)
- Acetylcholine chloride ≥99% (TLC) (Merck, Darmstadt, Germany)

- N-acetyl-D-phenylalanine> 98.0% (HPLC) (TCI, Boereveldseweg, Belgium)
- Uric acid ≥99% (Merck, Darmstadt, Germany)
- 1-methyluric acid (Sigma-Aldrich, Steinheim, Germany)

Standards used for the "Mercapturic acid mixture"

- N-acetyl-S-(2-hydroxypropyl) cysteine dicyclohexylammonium salt (2-HPMA) (Toronto Research Chemicals, Ontario, Canada)
- N-acetyl-S-(3-hydroxypropyl) cysteine dicyclohexylammonium salt (3-HPMA) (Toronto Research Chemicals, Ontario, Canada)
- N-acetyl-S-(carbamoylethyl)-L-cysteine (AAMA) (Toronto Research Chemicals, Ontario, Canada)
- N-acetyl-S-(N-methylcarbamoyl)-L-cysteine (AMCC) (Toronto Research Chemicals, Ontario, Canada)
- N-Acetyl-S-(2-cyanoethyl)-L-cysteine ammonium salt (CEMA) (Toronto Research Chemicals, Ontario, Canada)
- N-acetyl-S-(3-carboxy-2-propyl)-L-cysteine disodium salt, mixture of diastereomers (CMEMA) (Toronto Research Chemicals, Ontario, Canada)
- N-acetyl-S-(3,4-dihydroxybutyl)-L-cysteine, mixture of diastereomers (DHBMA) (Toronto Research Chemicals, Ontario, Canada)
- N-acetyl-S-ethyl-L-cysteine (EMA) (Toronto Research Chemicals, Ontario, Canada)
- N-acetyl-S-(2-hydroxy-3-propionamide)-L-cysteine dicyclohexylammonium salt (GAMA) (Toronto Research Chemicals, Ontario, Canada)
- N-acetyl-S-(2-hydroxyethyl)-L-cysteine dicyclohexylammonium salt (HEMA) (Toronto Research Chemicals, Ontario, Canada)
- N-acetyl-S-(3-hydroxypropyl-1-methyl)-L-cysteine dicyclohexylammonium salt, mixture of diastereomers (HMPMA) (Toronto Research Chemicals, Ontario, Canada)
- (R, S)-N-acetyl-S-[1-(hydroxymethyl)-2-propen-1-yl]-L-cysteine + (R, S)-N-acetyl-S-(2-hydroxy-3-buten-1-yl)-L-cysteine, 1:1 mixture (MHBMA) (Toronto Research Chemicals, Ontario, Canada)
- N-acetyl-S-methyl-L-cysteine (MMA) (Toronto Research Chemicals, Ontario, Canada)
- S-(4-nitrophenyl) mercapturic acid (NANPC) (Toronto Research Chemicals, Ontario, Canada)
- N-acetyl-S-(2-hydroxy-1-phenylethyl)-L-cysteine + N-acetyl-S-(2-hydroxy-2-phenylethyl)-L-cysteine, mixture (PHEMA) (Toronto Research Chemicals, Ontario, Canada)
- N-acetyl-S-benzyl-L-cysteine (SBMA) (Toronto Research Chemicals, Ontario, Canada)
- N-acetyl-S-phenyl-L-cysteine (SPMA) (Tokyo Chemical Industry, Tokyo, Japan)

Standards used for the "mixture of phthalates"

- Bisphenol A (BPA) 98% (Toronto Research Chemicals North York, Canada)
- Monobenzyl phthalate (MBzP) 98% (Toronto Research Chemicals North York, Canada)

- Mono-2-ethyl-5-carboxypentyl phthalate (MECPP) 95% (Toronto Research Chemicals North York, Canada)
- Monoethyl phthalate (MEP) 98% (Toronto Research Chemicals North York, Canada)
- Monoisobutyl phthalate (MiBP) 98% (Toronto Research Chemicals North York, Canada)
- Mono-n-butyl phthalate (MnBP) 98% (Toronto Research Chemicals North York, Canada)
- Mono-2-ethyl-5-carboxypentyl terephthalate (MECPTP) ≥95% (Cansin Chem Corp. Toronto, Canada)
- Mono-2-ethyl-5-hydroxyhexyl terephthalate (MEHHTP) ≥95% (Cansin Chem Corp. Toronto, Canada)

Commercial kits

- “Biocrates commercial QCH kit” (Biocrates Life Sciences AG, Innsbruck, Austria),
- kit used for targeted metabolomics analysis containing 188 standards. QCH is the highest concentration quality control of the most representative metabolites
- "Endocrine Urine Control Pathological Range" (Chromsystems, Gräfelfing, Germany)
- "Mixture for Occupational Medicine" (Recipe Chemicals + Instruments GmbH, München, Germany)

Other standards

- *o*-methylhippuric acid (TCI, Tokyo, Japan)
- *m*-methylhippuric acid (TCI, Tokyo, Japan)
- *p*-methylhippuric acid (TCI, Tokyo, Japan)
- N-acetylcysteine (Sigma-Aldrich, Steinheim, Germany)

Dissolution of analytical standards

- 1-hydroxypyrene was dissolved in acetonitrile to obtain a concentration of 0.1 mg/mL.
- Cortisone was dissolved in an acetonitrile:methanol mixture (1:1 v/v) to obtain a concentration of 0.1 mg/mL.
- 8-hydroxyguanosine, 8-hydroxy-2'-deoxyguanosine, and 8-hydroxyguanine were dissolved in water to obtain a concentration of 0.1 mg/mL each.
- Cotinine and N-acetylcysteine were dissolved in water to obtain a concentration of 0.1 mg/mL each.
- 3-methyluric acid, 1,7-dimethyluric acid, and 1-methyluric uric acid were dissolved in an ammonia solution to obtain a concentration of 0.1 mg/mL each.
- Ethylglucuronide and dexamethasone (Decadron®) were dissolved in methanol to obtain a concentration of 0.1 mg/mL each.
- *o*-metilippuric acid, *m*-metilippuric acid, and *p*-metilippuric acid, 1-naphthol, 3,5,6-trichloro-2-pyridinol, nicotine, 2-naphthol, aldosterone, cortisol, prednisone, prednisolone, ethylenediourea, ethyl sulfate, urea, dimethylthiourea, propylene thiourea, thiourea, benzoic acid, 3,4-dimethilippuric acid, 3-benzoylpropionic acid, 5-

(N-benzoylamino)-n-valeric acid, acetylcholine, and N-acetyl-D-phenylalanine were dissolved in methanol to obtain a concentration of 1 mg/mL each

- *Trans,trans*-muconic acid was dissolved in a phosphate buffer to obtain a concentration of 0.1 mg/mL.
- Each standard of the "mercapturic acid mixture" was dissolved in methanol to obtain a concentration of 1 mg/mL each, except CMEMA (concentration of 0.5mg/mL), and SBMA (concentration of 0.1 mg/mL).
- Each standard of the "mixture of phthalates" was dissolved in methanol to obtain a concentration of 1 mg/mL.

Preparation of standard mixtures

Standard mixture A

200 µL were taken from:

- 8-hydroxyguanosine 0.1 mg/mL in water
- 8-hydroxy-2'-deoxyguanosine 0.1 mg/mL in water
- 8-hydroxyguanine 0.1 mg/mL in water

then, 1400 µL of the urine mixture was added, to obtain a concentration of 10 µg/mL of each compound. Afterwards, a further dilution was performed by adding 100 µL of this solution to 900 µL of the urine mixture, to obtain a concentration of 1 µg/mL of each compound.

Standard mixture B

20 µL were taken from:

- 1-naphthol 1 mg/mL in methanol
- 3,5,6-trichloro-2-pyridinol 1 mg/mL in methanol
- Cotinine 1 mg/mL in water
- Nicotine 1 mg/mL in methanol

200 µL were taken from:

- 1-hydroxypyrene 100 µg/mL in acetonitrile

Then, 1720 µL of the urine mixture was added, to obtain a concentration of 10 µg/mL of each compound. Afterwards, a further dilution was performed by adding 100 µL of this solution to 900 µL of the urine mixture, to obtain a concentration of 1 µg/mL of each compound.

Standard mixture C

20 µL were taken from:

- 2-naphthol 1 mg/mL in methanol
- Aldosterone 1 mg/mL in methanol
- Cortisol 1 mg/mL in methanol
- Prednisolone 1 mg/mL in methanol

200 µL were taken from:

- Cortisone 100 µg/mL in acetonitrile:methanol (1:1)
- Dexamethasone 100 µg/mL in methanol
- Prednisone 100 µg/mL in methanol

Then, 1320 µL of the urine mixture was added, to obtain a concentration of 10 µg/mL of each compound. Afterwards, a further dilution was performed by adding 100 µL of this solution to 900 µL of the urine mixture, to obtain a concentration of 1 µg/mL of each compound.

Standard mixture D
20 µL were taken from:

- Ethylentiourea 1 mg/mL in methanol
- Ethylsulfate 1 mg/mL in methanol

200 µL were taken from:

- Ethylglucuronide 100 µg/mL in methanol
- *Trans,trans*-muconic acid 100 µg/mL in phosphate buffer

Then, 1360 µL of the urine mixture was added, to obtain a concentration of 10 µg/mL of each compound. Afterwards, a further dilution was performed by adding 100 µL of this solution to 900 µL of the urine mixture, to obtain a concentration of 1 µg/mL of each compound.

Standard mixture E
20 µL were taken from:

- Benzoic acid 1 mg/mL in methanol
- Dimethylthiourea 1 mg/mL in methanol
- Propylentiourea 1 mg/mL in methanol
- Thiourea 1 mg/mL in methanol
- Urea 1 mg/mL in methanol

200 µL were taken from:

- 3-methyluric acid 100 µg/mL in ammonia solution
- 1,7-Dimethyluric acid 100 µg/mL in ammonia solution

Then, 1500 µL of the urine mixture was added, to obtain a concentration of 10 µg/mL of each compound. Afterwards, a further dilution was performed by adding 100 µL of this solution to 900 µL of the urine mixture, to obtain a concentration of 1 µg/mL of each compound.

Standard mixture F
20 µL were taken from:

- Acetylcholine 1 mg/mL in methanol
- 3-benzoylpropionic acid 1 mg/mL in methanol
- 3,4-Dimethylhippuric acid 1 mg/mL in methanol
- 5-(N-benzoylamino)-*n*-valeric acid 1 mg/mL in methanol
- N-acetyl-D-phenylalanine 1 mg/mL in methanol

200 µL were taken from:

- 1-methyluric acid 100 µg/mL in ammonia
- Uric acid 100 µg/mL in ammonia

Then, 1500 µL of the urine mixture was added, to obtain a concentration of 10 µg/mL of each compound. Afterwards, a further dilution was performed by adding 100 µL of this solution to 900 µL of the urine mixture, to obtain a concentration of 1 µg/mL of each compound.

Other standards

The solutions of *o*-metilippuric, *p*-metilippuric, *m*-metilippuric and N-acetylcysteine at 1 mg/mL were separately diluted with the urine mixture to obtain a final concentration of 1 µg/mL (2 µL of each was added to 1998 µL of the urine mixture).

Mercapturic acid mixture

The standard solutions of mercapturic acids were then diluted in synthetic urine to a final concentration of 0.04 mg/L for EMA, MHBMA, MMA, NANPC, PHEMA, SBMA, and SPMA, 1.6 mg/L for 2-HPMA, 3-HPMA, AAMA, CEMA, DHBMA, GAMA, and HEMA and 3.2 mg/L for AMCC, CMEMA and HMPMA.

Mixture of phthalates

These solutions were then diluted in water to a final concentration of 0.05 mg/L for BPA, 0.1 mg/L for MBzP, MECPP, MECPTP and MEHHTP and 0.5 mg/L for MEP, MiBP and MnBP. 100 µL of this solution was added to 900 µL of the urine mixture to further dilute the solution.

Commercial kits

Commercial kits were prepared with water as reported in the related instructions, in particular.

- “Biocrates - QCH”: 100 µL of water was added, and it was then shaken at 1200 rpm for 15 min. Then 50 µL of the freshly prepared solution was diluted in 450 µL of the urine mixture.

- "Chromsystem - Endocrine Urine Control Pathological Range": 8 mL of water was added, the solution was then left to rest for 10-15 min and then shaken to dissolve the contents.

- “Recipe - For Occupational Medicine”: 5 mL of water was added, and it was shaken for 15 min.
